# Effectiveness of patient feedback as an educational intervention to improve medical student consultation (PTA Feedback Study): study protocol for a randomized controlled trial

**DOI:** 10.1186/1745-6215-15-361

**Published:** 2014-09-17

**Authors:** Michelle Mei Yee Lai, Noel Roberts, Jenepher Martin

**Affiliations:** Medical Student Programs, Eastern Health Clinical School, Monash University, Faculty of Medicine, Nursing and Health Science and Deakin University, School of Medicine, Level 3, 5 Arnold Street, Box Hill, Victoria, 3128 Australia

**Keywords:** Communication skills, consumer feedback, medical education, medical students, multisource feedback, patient satisfaction, professionalism, teaching

## Abstract

**Background:**

Oral feedback from clinical educators is the traditional teaching method for improving clinical consultation skills in medical students. New approaches are needed to enhance this teaching model. Multisource feedback is a commonly used assessment method for learning among practising clinicians, but this assessment has not been explored rigorously in medical student education. This study seeks to evaluate if additional feedback on patient satisfaction improves medical student performance.

**Methods:**

The Patient Teaching Associate (PTA) Feedback Study is a single site randomized controlled, double-blinded trial with two parallel groups.

An after-hours general practitioner clinic in Victoria, Australia, is adapted as a teaching clinic during the day. Medical students from two universities in their first clinical year participate in six simulated clinical consultations with ambulatory patient volunteers living with chronic illness. Eligible students will be randomized in equal proportions to receive patient satisfaction score feedback with the usual multisource feedback and the usual multisource feedback alone as control. Block randomization will be performed. We will assess patient satisfaction and consultation performance outcomes at baseline and after one semester and will compare any change in mean scores at the last session from that at baseline. We will model data using regression analysis to determine any differences between intervention and control groups. Full ethical approval has been obtained for the study. This trial will comply with CONSORT guidelines and we will disseminate data at conferences and in peer-reviewed journals.

**Discussion:**

This is the first proposed trial to determine whether consumer feedback enhances the use of multisource feedback in medical student education, and to assess the value of multisource feedback in teaching and learning about the management of ambulatory patients living with chronic conditions.

**Trial registration:**

Australian New Zealand Clinical Trials Registry (ANZCTR): ACTRN12613001055796.

## Background

Multisource feedback is a strong motivator in modifying clinicians’ behaviour and promoting reflective practice [1, 2]. Feedback may facilitate more accurate assessment of clinicians’ own skills by providing them with information that they may have unintentionally overlooked or underemphasized, and by identifying problems that could jeopardize patient satisfaction in a clinical consultation [3, 4].

Routine feedback to clinicians has been shown to improve client outcomes at the end of trainees’ practicum training, compared with no feedback [4, 5]. Although multisource feedback is the accepted workplace assessment of professional behaviours in training doctors [6–8], this form of assessment and feedback is not commonly integrated into medical student education. Central to this multisource feedback model is direct, immediate feedback from patients, peers and tutors to students, formally integrating multisource feedback into the teaching episode.

Interpersonal and communication skills in clinical consultations have been identified as a core competency in physicians, because adequate skills could enhance patient satisfaction, therapy compliance, symptom relief and cost effectiveness [9, 10]. The traditional mode of teaching these skills in ambulatory care is oral feedback from educators and peers on student consultation skills and professionalism. The effectiveness of written feedback from patients in medical student education has not been vigorously explored [6, 7, 11].

### Objectives and hypothesis

This study aims to examine whether additional patient satisfaction feedback to medical students after ambulatory consultations improves the medical students’ clinical consultation performance. We hypothesize that additional written feedback from patients to students, in the form of completed MISS-21 questionnaires, would improve both student performance in patient satisfaction scores (primary outcome) and clinical consultation skills, as reported by tutors (secondary outcome); and that the multisource feedback model increases patient satisfaction outcome in student consultations over time.

## Methods/design

### Study design

**Figure 1:**
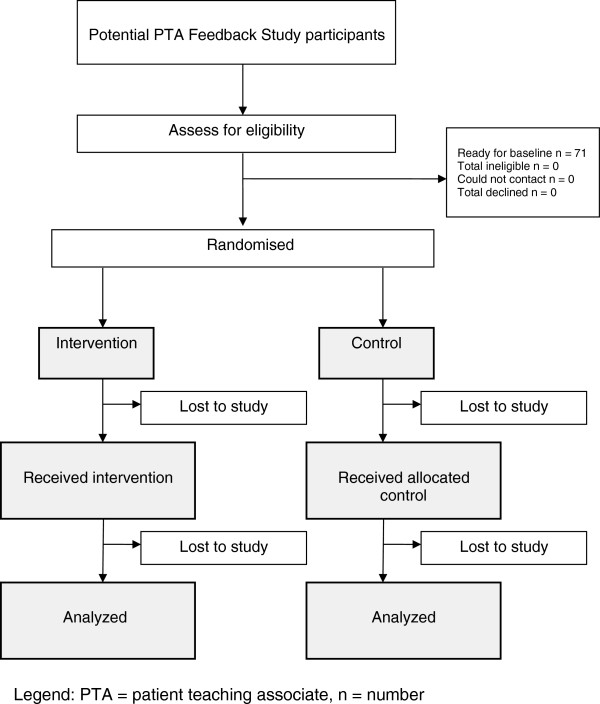
**Flow chart of the proposed Patient Teaching Associate (PTA) feedback study.** PTA, patient teaching associate.

The Patient Teaching Associate (PTA) Feedback Study is designed as a randomized, controlled, assessor- and patient-blinded, single-centre exploratory trial with two parallel groups after six student consultation sessions (Figure 1). The CONSORT statement has been used as the framework for the methodology of this study.

### Setting and participants

The trial will be conducted from March 2013 to March 2014 at Monash University Eastern Health Clinical School, Victoria, Australia. The setting of the teaching programme is an after-hours general practitioner clinic that is adapted as a medical student teaching clinic during the day. The programme recruits real patients with chronic illnesses from the community, and aims to promote a patient-centred approach to consultation [12]. Clinical tutors are senior medical practitioners, including general practitioners, physicians and surgeons.

Sixty-six medical students in their first clinical year will be recruited to this study. Participants eligible for the trial are third-year students enrolled in Monash University and Deakin University attending Eastern Health Clinical School and willing to participate in the teaching programme. Students will see the same patients in groups of three. There are no exclusion criteria. Written consent will be obtained from all participants.

### Interventions

Eligible students will be randomized in equal proportions to receive either patient satisfaction score feedback with usual verbal multisource feedback or usual verbal multisource feedback alone, as control.

Students in both the intervention and control group receive oral feedback from the tutor, PTA and peers immediately following each patient consultation. Both intervention and control groups receive immediate oral feedback according to the Rating Instrument of Clinical Consulting Skills (RICS) framework [13].

The educational intervention is written feedback using the 21-item Medical Interview Satisfaction Scale (MISS-21) in addition to the usual oral feedback [14]. The MISS-21 is a widely available 21-item validated visit-based questionnaire for measuring patient satisfaction in the primary care context. The MISS-21 questionnaire can be found in appendix 1 of the article by Meakin *et al.* [14]. Students in the intervention group will receive their intervention pack no later than one week prior to the last consultation during one semester (generally six consultations in total). The intervention pack includes patient feedback questionnaires for all previous student-led consultations as well as written instructions about self-reflection on the feedback received based on the Pendleton feedback framework [15].

### Primary outcome measure

The primary outcome measure is patient satisfaction immediately after the student consultation episodes, rated using the MISS-21 [14]. The consultation satisfaction questionnaire has been used to rate general practitioners and nurse practitioners. It is chosen because of its ease of administration, as it is visit-based and free from cost- or facility-based questions, has reported validity and reliability, and is a commonly used feedback tool in the consultation based clinical setting [16–18]. An independent researcher interviews each PTA after each consultation to obtain the scores.

### Secondary outcome measures

The RICS is the secondary outcome measure. The performance score by tutors gives a composite score and subscale scores of patient-centred approach, history taking, physical examination and problem solving and management. Tutors receive standardization training based on a video and complete the assessments within 24 hours of the student consultation episodes. The RICS was chosen because it is a student performance assessment tool designed for the Patient Partnership Program, a similar teaching programme developed at the Launceston Clinical School, University of Tasmania [19, 20]. Its construct validity and psychometric properties have been reported [13]. The concurrent use of the RICS avoids the risk of a simple training effect on MISS-21 scores.

### Sample size

In the power calculation, we used the unpaired *t* test to detect a difference in the primary outcome (MISS-21) between the two groups. The RICS score, as the secondary outcome measure, was not used in the power calculation.

We incorporated the standard deviation in a nurse practitioner group in a trial using MISS-21 measurement [21]. There is, to date, no student data on MISS-21, and nurse practitioners are therefore chosen as the most likely comparator group. The medical student participants, in their first clinical year, are more likely still to be using a â€˜script-based’ clinical consultation style with less variability and are not considered comparable to experienced doctors. With 33 participants per group, there is 80% power of detecting a difference of at least 0.32 points in the MISS-21 at 5% significance level, assuming the standard deviation in the control group is 0.46 [21]. Cohen’s *d* = 0.69 indicates that a difference of 0.32 points has a moderate to large effect size in the primary outcome.

### Randomization

Assignment of interventions will be by block randomization, according to a list of computer-generated random numbers.

### Allocation concealment and blinding

Allocation numbers are kept in sealed containers. Tutor and patient assessors will be blinded to group membership. Emergency unblinding will be considered only on student participants’ request and approval. For example, a student may experience significant distress and request tutor counselling or debriefing after receiving written feedback.

Tutor assessors, patient assessors and data analysts are blinded to group assignment of participants. Because of the nature of the feedback, student participants will not be blinded to the group membership.

### Implementation

An investigator (ML) generates the allocation sequence using computer-generated numbers and conceals the random sequence in sealed opaque envelopes. Another investigator (NR), not directly involved in the assessment of students, draws the envelopes and assigns participants to their study groups.

Student participants are required to complete a common assessment battery at the time of enrolment: demography, baseline RICS and MISS-21 after their first consultation. At the end of baseline assessment, student participants are randomly allocated to the RICS group (control) or combining RICS and MISS-21 (intervention).

Students in the intervention group will be able to obtain MISS-21 patient satisfaction feedback no later than one week prior to the final consultation session near the end of the semester. They are requested to complete a reflection exercise using the Pendleton model [15]. Students in the control group will be able to obtain patient feedback using the MISS-21 within one month following their final consultation session (after the intervention period). Hence, the control group will still have an opportunity for self-reflection and benefit from patient feedback to improve their consultation skills.

### Adherence

We will recruit third-year medical students in their first clinical year to participate in the trial. This group of students is highly motivated in learning from real patient volunteers. A very high rate of retention and adherence in participation is expected.

Email adherence reminders will be sent after the participants have obtained written feedback of patient satisfaction. This reminder will emphasize the importance of following study guidelines to read the written feedback and the importance of contacting the coordinator if experiencing problems related to the study intervention. Debriefing and referral for counselling will be available to any student upon request.

### Data management and statistical methods

All data will be entered electronically. The dataset will be recorded in a spreadsheet maintained on a secured University server. All forms related to the study will be kept in locked cabinets. Access to the study forms and electronic data will be restricted. All reports will be prepared such that no individual participant can be identified.

All data will be analyzed using SPSS (IBM Corp. Armonk, NY, USA.) and Stata (StataCorp LP, College Station TX, USA). We will compare student characteristics between the two groups using a chi-squared test for categorical variables and a *t* test for continuous variables. We will model data using regression analysis to assess any change in mean score on the MISS-21 and RICS patient-centeredness subscale (dependent variables) at the first and last tutorial session for both groups and to determine whether any differences exist between intervention and control groups (independent variables), taking the following covariates into account: age, sex, education in years, postgraduate status and international student status. They may enter the regression model only if the covariates are imbalanced between the two groups after randomization due to chance.

Additional subgroup analyses will be performed for the following variables: undergraduate versus postgraduate study, work experience, local versus international students status and language spoken at home.

There will be no control group for patient assessors. We will use linear mixed effects regression to account for random effects of variability in student grouping and variability in patient characteristics.

### Ethics

This protocol has received ethical approval from the Monash University Human Research Ethics Committee project number CF13/779 - 2013000356.

## Discussion

The results of this trial will inform educators whether multisource feedback with and without patient satisfaction feedback can improve student performance from baseline assessment in the intervention and control group respectively, and whether the educational intervention is effective in improving patient outcome and student performance compared with participants in the control group. This educational intervention will be replicable to other tertiary institutions.

## Trial status

The trial is recruiting participants by invitation only.

## Authors’ information

MMYL: MBBS, MPH, GCHPE, FRACP, Lecturer, Medical Education Fellow at Medical Student Programs, Eastern Health Clinical School, Monash University and Deakin University, a practising consultant geriatrician and clinical epidemiologist.

NR: BSc (Hons), DipNutrFoodSci, CertDiet, GradDipHlthAdmin, Senior Lecturer, Medical Student Programs, Eastern Health Clinical School, Monash University and Deakin University, curriculum developer and PTA programme manager.

JM: MBBS, MEd, MS, DEd, FRACS, Associate Professor and Director, Medical Student Programs, Eastern Health Clinical School, Monash University and Deakin University and a practising consultant surgeon.

## Abbreviations

MISS-21: 21–item Medical Interview Satisfaction Scale
PTA: patient teaching associate
RICS: Rating Instrument of Clinical Consulting Skills
SPSS: Statistical Package for Social Sciences.
